# Greater pre-operative anxiety, pain and poorer function predict a worse outcome of a total knee arthroplasty

**DOI:** 10.1007/s00167-016-4314-8

**Published:** 2016-10-12

**Authors:** Sharifah Adla Alattas, Toby Smith, Maria Bhatti, Daniel Wilson-Nunn, Simon Donell

**Affiliations:** 1 Faculty of Medicine and Health Sciences, University of East Anglia, Norwich, UK; 2 Department of Orthopaedic Surgery, Norfolk and Norwich University Hospital, University of East Anglia, Norwich, UK; 3 Department of Statistics, University of Warwick, Coventry, UK

**Keywords:** Total knee arthroplasty, Depression, Anxiety, Results, Outcome, Quality of life

## Abstract

**Purpose:**

Around 10–30 % of patients are dissatisfied with the results of their total knee arthroplasty (TKA). This review aimed to identify and evaluate the predictors of outcome measured by the three domains of health-related quality of life (pain, stiffness and function). The focus was on pre-operative psychological factors as related to other patient-related variables.

**Methods:**

A systematic search was performed using the following databases: MEDLINE, PubMed, AMED, CINAHL, PsychINFO, SciFinder, Scopus, EMBASE, Cochrane, Lilacs, Web of Science and ScienceDirect. The quality of identified studies was assessed using the Critical Appraisal Skills Programme Cohort checklist.

**Results:**

Ten studies met the eligibility criteria. From these, nine patient-related predictors of outcome were identified (depression, anxiety, age at surgery, gender (being female), medical co-morbidities, BMI, level of education, pre-operative pain severity and pre-operative knee function). Greater anxiety, pre-operative pain and function were the most significant factors to predict a poorer outcome of a TKA. The results of depression, gender (female), medical co-morbidities, BMI and level of education were variable among the included studies. There was very little evidence to support older age at operation as a predictor of poorer outcome.

**Conclusion:**

Patients experiencing high levels of pain before surgery should be informed of the chances of improvement by having a TKA. A validated psychological screening tool that separates depression and anxiety is recommended as part of the pre-operative assessment stage. Patients presenting with symptoms of depression and anxiety should be identified and consulted before a TKA.

**Level of evidence:**

II.

**Electronic supplementary material:**

The online version of this article (doi:10.1007/s00167-016-4314-8) contains supplementary material, which is available to authorized users.

## Introduction

To date, the preferred therapeutic option for advanced osteoarthritis (OA) of the knee is a ‘total knee arthroplasty’. The current method of evaluating the results of surgery may be unsatisfactory in exploring the patient’s attitude towards the outcome of surgery [28]. Patient-reported outcome measure (PROM) questionnaires have recently been implemented to evaluate a patient’s quality of life (QoL) after surgery [18].

Despite the surgical success of total knee arthroplasty (TKA), about 20 % of all patients are dissatisfied [27]. Numerous studies suggest that a suboptimal post-operative result is associated with chronic knee pain and lower than expected function [21, 22, 26]. Others attribute patient-related factors such as gender (being female), a higher body mass index and depression to an increased risk of post-operative pain and stiffness [8].

Approximately 30 % of patients who undergo a TKA experience pre-operative psychological distress [1]. Patients who are deemed anxious or depressed are reported to have a worse outcome score in comparison with those with a better psycho-social component score [12, 16]. The degree to which anxiety and depression affect outcome when compared to other patient-related factors is unclear.

The purpose of this study was to identify and evaluate the most common predictors of outcome using the domains of HRQoL (pain, stiffness and function) [6] with a particular emphasis on psychological factors.

It is anticipated that this study will add to existing knowledge by identifying patient-related variables that predict a worse outcome. Subsequently, new recommendations can be made to improve the HRQoL of these patients before and after TKA in order to reduce the rate of dissatisfaction after surgery.

## Materials and methods

### Search strategy

All published studies from its inception to 18 January 2016 were searched. This included: MEDLINE, PubMed, AMED, PsychINFO, CINAHL, SciFinder, ScienceDirect, EMBASE, Cochrane, Scopus, Web of Science and Lilacs. The reference lists of all potentially eligible studies were reviewed and, where necessary, corresponding authors were contacted. A list of search terms and Boolean operators were used (Supplementary Table 1). The search was limited to ‘full-text’ articles and studies on ‘human’ subjects.

### Study selection

Two reviewers independently selected the papers to be included in this review (AA and MB). This review was limited to primary studies without linguistic or geographic constraints; non-English language papers were included. The search was not limited to publication date or location of publication. Disagreements on paper eligibility were resolved through discussion or consulting with the appointed adjudicator (DWN).

### Eligibility criteria

All articles that met the following criteria were included that:

1. Compared pre-operative against post-operative results.
2. Included one or more outcome measures for depression and anxiety.
3. Evaluated the effects of both depression and anxiety as a predictive variable for the outcome of TKA.
4. Used validated outcome measures.
5. Had osteoarthritis as the primary reason for surgery in more than 90 % of participants.
6. Had the first follow-up period within 6 months of surgery.
7. If in a foreign language, had a published translated copy is made available in English.

The following were excluded from the review:

1. Studies that failed to separate results of patients who underwent a TKA and those that had evaluated alternative surgical procedures.
2. A comparison was not made between pre-operative and post-operative results.
3. Papers that focused on either depression or anxiety.
4. Articles that aimed to compare or validate outcome measures.
5. Studies that involved retrospective questionnaires.

### Data extraction and critical appraisal

Data were extracted from each included paper on country of origin; method of recruitment; number of participants age; gender; TKA procedure; characteristics of the participants; outcome measures; ethical approval; length of follow-up period; number of follow-up periods and main findings of the study. Using the modified ‘Cochrane Data Collection Form’, data were independently extracted from the included papers by two reviewers (AA and MB). Thereafter, the data were assessed for any inconsistencies. Two reviewers (AA and MB) independently assessed the methodological quality of each paper using a modified ‘Critical Appraisal Skills Programme (CASP) Cohort Study Checklist’. This checklist consists of 12 questions that evaluated the validity, consistency and reliability of each study [14]. The results were then interpreted in the context of the research question.

The data accuracy was calculated by the adjudicator (DWN), using kappa coefficient to measure inter-rater reliability [17]. Any disagreements were resolved through consensus between the reviewers.

### Outcome measures

The primary outcome was the HRQoL of each patient after a TKA. The definition of HRQoL was devised by the domains analysed in the patient-reported outcome measure (PROM) questionnaires for patients of the National Health Service [11, 18]. This is linked to the National Joint Registry and includes the Oxford Knee Score and the EuroQol Index score [2]. The PROM programme requires data to be collected of condition-specific outcomes such as pain, stiffness and function, before and at 6 months after a TKA. HRQoL includes various domains such as post-operative stiffness, pain and function [6]. All HRQoL outcome measures that analysed the effects of pain, function or stiffness were included as potential outcome measures in this review. The secondary outcome was the effect of depression and anxiety as a predictive factor when compared to other patient-related variables. The psychological variables that were assessed in this study are anxiety and depression. The two variables are potential predictive psychological variables of a poor outcome [3, 24, 29]. Further, common psychological outcome measures such as the ‘Hospital Anxiety and Depression scale’ are developed to detect the effects of the aforementioned variables.

### Data analysis

Each paper was analysed to identify significant results about each predictor variable. Nine variables were assessed: pre-operative pain, pre-operative function, depression, anxiety, gender, education level, body mass index (BMI), age at surgery and medical co-morbidities. Due to the limited data for individual predictors of interest, there were insufficient data to pool the results in a meta-analysis. Accordingly, a narrative analysis was employed. A sensitivity analysis was conducted in response to papers that provided unclear evidence to fulfil the pre-defined eligibility criteria.

## Results

### Summary of study characteristics

In total 1485 patients with 1518 TKAs were reported (Table 1). There were 576 male and 909 female patients with a mean age of 68 years (range 36 [5] to 92 [9]). The number of follow-up time points ranged from 1 to 7 from 1 month [5, 23] to 120 months after operation [31]. Five studies had a follow-up at 6 months [4, 5, 15, 20, 23]. All 10 studies were deemed as level 1B.

**Table 1:** Study characteristics

References	Study size/number of TKA	M:F	Mean age	Outcome measure	Psychological tool	Relevant independent variables	Study design
Blackburn et al. [4]	40/40	16:24	72 (52–88)	OKS	HAD	Depression; anxiety	Prospective cohort
Brander et al. [5]	116/149	52:64	66 (36–85)	VAS, MPQ; KSS; WOMAC	BDI; STAI	Depression; anxiety	Prospective observational cohort
Duivenvoorden et al. [7]	128/128	56:72	66.2 (56.5–75.9)	KOOS	HAD	Depression; anxiety	Prospective cohort
Hanusch et al. [9]	100/100	55:45	71 (42–92)	OKS	HAD	Depression; anxiety	Prospective cohort
Hirschmann et al. [10]	104/104	46:58	70 (59–81)	KSS, WOMAC	BDI, STAI	Depression; anxiety	Prospective cohort
Lopez-Olivo et al. [15]	241/241	78:163	65 (56–74)	WOMAC; KSRS	DASS-D; DASS-A	Depression; anxiety; age at surgery; gender; medical co-morbidities; BMI; education	Prospective cohort
Qi et al. [23]	119/119	38:81	62.1 (51.98–72.22)	KSS; SF-36	STAI; BAI; BDI; TAI; SAI	Depression; anxiety	Prospective cohort
Noiseux et al. [20]	215/215	90:125	61.98 (52.16–71.8)	ROM	STAI; GDS	Depression; anxiety; age at surgery; gender; BMI; education	Prospective cohort
Utrillas-Compaired et al. [30]	202/202	62:140	73 (SD 6.35)	KSS; WOMAC; VAS	HAD	Depression; anxiety; age at surgery; BMI	Prospective cohort
Wylde et al. [31]	220/220	83:137	70 (SD 9)	WOMAC	HAD	Depression; anxiety; age at surgery; gender; medical co-morbidities	Prospective cohort

The majority of studies had a clear distinction between psychological assessments and general outcome tools. In total, there were nine outcome measures and nine separate psychological tools. The WOMAC (Western Ontario and McMaster University Osteoarthritis Index) scale [7, 10, 15, 30, 31] and the HAD (Hospital Anxiety and Depression) score [4, 7, 9, 30, 31] were the most frequently used measures.

### Critical appraisal assessment

The evidence presented with a number of strengths (Supplementary Table 2); for example, each included study had a focused objective. Three papers [4, 20, 23] failed to identify a gap in the existing literature. All outcome measures were validated. A number of studies acknowledged confounding factors in the design and/or analysis. Four papers [7, 15, 20, 31] adjusted for potential confounders. The majority of papers [5, 7, 23, 30, 31] used regression analysis, while Lopez-Olivo et al. [15] used both regression and sensitivity analysis. All studies included in this review were deemed as high-quality research evidence. Further information on patient demographic factors in six studies [4, 5, 10, 23, 31] would improve the credibility of its results. There was a consistency in the results of all included papers apart from one [23], which could not be explained compared to the evidence base in its totality.

One paper [4] failed to provide sufficient details of the criteria used for the selection of subjects. Half of the included papers provided details on the socio-demographic characteristics of the participants. Participant attrition was a notable limitation as illustrated in Table 2.

**Table 2:** Results of data analysis

Study and year	Loss to follow-up rate (%)	Reported follow-up time (months)	Reported follow-up time points (months)
Blackburn et al. [4]	NI	6	3 and 6
Brander et al. [5]	NI	12	1, 3, 6 and 12
Duivenvoorden et al. [7]	39	12	3 and 12
Hanusch et al. [9]	13	12	1.5 and 12
Hirschmann et al. [10]	5	12	1.5, 4 and 12
Lopez-Olivo et al. [15]	11	6	6
Qi et al. [23]	12	6	1 and 6
Noiseux et al. [20]	NI	6	6
Utrillas-Compaired et al. [30]	23	12	12
Wylde et al. [4]	12	12	12

*NI* not included

Inter-rater agreement was calculated by the adjudicator (DWN). This resulted in a kappa value of 0.795, indicating substantial agreement [13].

### Clinical predictive factors

#### Depression

Three studies [5, 10, 23] evaluated the effects of depression as a predictor using the Beck Depression Scale (BDI) (Supplementary Table 3). Using the BDI, Brander et al. [5] demonstrated that patients with higher levels of depression suffered from a greater severity of pain at 1 year after surgery. Hirschmann et al. [10] reported pre-operative depression (using BDI), predicted worse pain (Spearman’s rho = 0.29; *p* < 0.05), greater stiffness (Spearman’s rho = 0.34; *p* < 0.01) and poorer function (Spearman’s rho = 0.37; *p* < 0.001) using WOMAC at 1 year. Using the KSS, both Qi et al. [23] and Hirschmann et al. [10] did not identify a significant association between depression, greater post-operative pain and poorer function at 6 months after surgery. Perhaps, baseline depression would predict a worse outcome if different HRQoL outcome measures were used.

The majority of studies [4, 7, 9, 30, 31] used the HAD score to evaluate the effects of depression. Greater pre-operative depression correlated with higher levels of knee disability (coefficient—0.409; *p* = 0.009) [4] and a reduction in the change of pain [7]. Utrillas-Compaired et al. demonstrated that pre-operative depression had a significant influence on the change in function after surgery and QoL score at 1 year (beta coefficient—2.024; *p* = 0.028) [30].

Noiseux et al. [20] and Lopez-Olivo et al. [15] identified a correlation between pre-operative depression and higher levels of pain at 6 months after surgery. In addition, using multiple health-related outcome measures, it was found that a higher depression score predicted poorer function at 6 months [9, 15] and at 1 year after surgery [9].

Only Wylde et al. [31] did not find depression to predict a poorer post-operative WOMAC pain and function score.

#### Anxiety

The majority of studies [4, 9, 30, 31] identified pre-operative anxiety as a predictor of a poorer outcome using the HAD scale (Supplementary Table 4). Pre-operative anxiety levels were associated with higher levels of knee disability [4] and significant functional and pain limitations using WOMAC [30], including a worse quality of life (QoL) at 1 year [15]. However, the change in KSS function and pain was not attributable to pre-operative depression [30].

Pre-operative anxiety did not predict a poorer post-operative ROM score and functional outcome at 6 weeks and 1 year [9]. However, pre-operative anxiety predicted a poorer OKS result at both time intervals [9]. Duivenvoorden et al. [7] demonstrated that pre-operative anxiety significantly predicted a worse QoL.

Three papers analysed anxiety using the State Trait Anxiety Inventory (STAI) score. Using the Visual Analogue Score (VAS) pain scale, Brander et al. [5] demonstrated that pre-operative anxiety strongly predicted pain at 1 year (*p* < 0.03). Hirschmann et al. [10] found that higher levels of pre-operative trait anxiety predicted greater pain (Spearman’s rho = 0.25; *p* < 0.05) and stiffness (Spearman’s rho = 0.26; *p* < 0.05). Qi et al. [23] demonstrated that pre-operative anxiety as measured by the Trait Anxiety Inventory (TAI) predicted poorer knee function at 6 months.

Lopez-Olivo et al. [15] found pre-operative anxiety, as measured by the Depression Anxiety Stress Scales Anxiety (DASS-A), predicted a worse WOMAC pain (Spearman’s rho = 0.17; *p* = 0.007), WOMAC function (Spearman’s rho = 0.18; *p* = 0.0001) and KSRS total (Spearman’s rho = −0.12; *p* = 0.002) scores.

The results of age at surgery, gender, medical co-morbidities, BMI, education, pre-operative pain and function are reported in Supplementary Table 5.

#### Age

Four studies [15, 20, 30, 31] analysed the effects of age on the outcome of a TKA.

Older age at surgery was associated with a worse total KSRS scores [15], but did not predict poorer pain [20, 31] and function [31] scores at 1 year. Utrillas-Compaired et al. [30] found that age at surgery significantly predicted a poorer post-operative KSS function score (beta coefficient—0.396; *p* = 0.004).

#### Gender

Lopez-Olivo et al. [15] found that being female was associated with decreased function using KSRS and with WOMAC pain (Spearman’s rho = 0.15; *p* = 0.02) and function (Spearman’s rho = 0.18; *p* = 0.004). However, Wylde et al. [31] found that gender did not significantly predict poorer WOMAC pain and function scores at 1 year. Noiseux et al. [20] found that gender did not predict post-operative pain with range of motion.

#### Medical Co-morbidities

Three studies [15, 30, 31] analysed the effects of co-morbidities. Lopez-Olivo et al. [15] observed that co-morbidity predicted a worse WOMAC pain (Spearman’s rho = 0.2; *p* = 0.002), WOMAC function (Spearman’s rho = 0.13; *p* = 0.05) and total KSRS (Spearman’s rho = −0.22; *p* = 0.001) score.

Utrillas-Compaired et al. [30] showed that a higher number of medical co-morbidities predicted a poorer functional outcome score. However, Wylde et al. [31] demonstrated that the number of co-morbidities did not affect the post-operative function or pain scores.

#### Body mass index

There were three studies [15, 20, 30] that explored the effects of BMI. Lopez-Olivo et al. [15] showed that a higher BMI score predicted worse WOMAC pain (Spearman’s rho = 0.21; *p* = 0.001), WOMAC function (Spearman’s rho = 0.2; *p* = 0.002) and total KSRS (Spearman’s rho = −0.33; *p* < 0.0001) scores. Utrillas-Compaired et al. [30] showed that a higher BMI (beta coefficient—0.202; *p* < 0.001) predicted a poorer functional outcome score. Noiseux et al. [20] noted that BMI was not associated with a worse post-operative pain score.

#### Educational attainment level

Lopez-Olivo et al. [15] reported that lower educational level (measured as less than high school) was associated with greater pain (Spearman’s rho = −0.23; *p* = 0.0003) and function (Spearman’s rho = −0.19; *p* = 0.004) as measured by WOMAC. Noiseux et al. [20] noted that the education level did not predict post-operative pain severity.

#### Pre-operative pain severity

All four studies [15, 20, 30, 31] demonstrated that pre-operative pain predicted at least one HRQoL domain (Supplementary Table 5). Lopez-Olivo et al. [15] observed that a greater baseline WOMAC pain score predicted a poorer WOMAC pain (Spearman’s rho = 0.21; *p* = 0.001), function (Spearman’s rho = 0.15; *p* = 0.02) and total KSRS score (Spearman’s rho = −0.18; *p* = 0.009) at 6 months. Noiseux et al. [20] found that a greater pre-operative pain severity predicted worse post-operative knee pain.

Utrillas-Compaired et al. [30] demonstrated that only pre-operative KSS pain (beta coefficient—0.834; *p* < 0.01) was associated with post-operative pain. Lastly, Wylde et al. [31] showed that a higher pain severity was associated with the variance in pain severity (regression coefficient = 0.183; *p* = 0.016).

#### Pre-operative knee function

Three studies [15, 30, 31] demonstrated that poorer pre-operative function is a predictor of a poorer outcome (Supplementary Table 5). Lopez-Olivo et al. [15] found that a worse pre-operative WOMAC function score predicted worse post-operative WOMAC pain (Spearman’s rho = 0.27; *p* < 0.0001), WOMAC function (Spearman’s rho = 0.26; *p* < 0.0001) and total KSRS (Spearman’s rho = −0.28; *p* < 0.0001) scores. Utrillas-Compaired et al. [30] reported that the greater the severity of pre-operative function the worse the post-operative KSS function. Wylde et al. [31] showed that a worse pre-operative functional ability predicted greater functional limitations after TKA.

### Sensitivity analysis

Three papers [4, 7, 30] were removed from the sensitivity analysis. This affected the interpretation of five predictors. Despite the exclusion of two papers [23, 30], anxiety remained a strong predictor of worse HRQoL score and depression remained as a significant predictor of outcome. This analysis slightly affected the degree of certainty that pre-operative pain and function reduce the HRQoL. The probability of medical co-morbidities predicting a poorer outcome was halved. This analysis reduced the effect of BMI as a predictor of a poor outcome. This analysis showed that age at surgery, gender and education did not predict a poorer outcome of a TKA.

Anxiety, depression and education were unaffected by the sensitivity analysis.

## Discussion

All 10 studies included in this review demonstrated that a TKA improved a patient’s quality of life in at least one outcome measure. The majority of studies [4, 5, 7, 9, 10, 15] identified that a higher baseline anxiety and depression level predicted a poorer outcome. A higher pre-operative knee pain and poorer function were consistent predictors of a worse HRQoL after surgery. The remainder of predictive factors (age at surgery, gender, co-morbidities, BMI and level of education) varied in their effects on the outcome of a TKA. Lopez-Olivo et al. [15] found older age, lower education, BMI and co-morbidities to be associated with poorer outcomes. However, Wylde et al. [31] did not identify older age at surgery, gender (being female) or a higher number of medical co-morbidities as a predictor of a poor outcome.

Bistolfi et al. [3] acknowledged that a significant number of patients undergoing a TKA suffer from subclinical depression. These patients are more likely to suffer from a greater pain severity at 1 year compared to those without signs of clinical depression. Judge et al. [12] produced similar results by suggesting that the strongest determinants of outcome are pre-operative pain, function, anxiety and depression. However, Nilsdotter et al. [19] reported that older age predicted more post-operative pain. A potential explanation is that these authors used all the five different KOOS subscales [19]. Additionally, Riddle et al. [25] did not find an association between psychological variables (anxiety and depression) and a poor WOMAC function outcome score. The chosen outcome measures included the Personal Health Questionnaire Depression scale (PHQ-8) and Generalised Anxiety Disorder and Panic Disorder modules from the PRIME-MD. The extent to which each potential predictive factor affects the outcome is complex due to the use of a variety of different PROMs.

This review found that pre-operative pain is a strong predictor of outcome, which implies that patients should be considered for an operation at an earlier stage when pain is less severe. Also, patients with high baseline pain score need advice about their chances of experiencing persistent pain, and strategies put in place to manage this.

Three papers analysed the effects of pre-operative function on outcome [15, 30, 31]. The results of this review demonstrate that pre-operative knee function is a strong predictor of functional outcome. This is consistent with the evidence from the existing literature. Therefore, it is suggested that surgery should not be delayed to allow the functional status of patients to deteriorate. It may be required that patients who score higher on their pre-operative questionnaires are prioritised for urgent surgery. However, further research should investigate the possibility of identifying a threshold score for surgery on the pre-operative questionnaires.

Further studies are required to evaluate the degree by which psychological factors affect the outcome when compared to other outcome measures. It may be that by treating pre-operative anxiety, as well as controlling pain and improving function, the quality of life for patients undergoing a TKA could be improved.

There are three key limitations in this review: first, the inability to pool the results together due to limited data available in each study and the variation in outcome measures used. Second, reporting bias may be present due to the use of multiple PROMs, clearly specifying three key HRQoL variables (function, pain and stiffness). Therefore, all included studies are conceptually similar as they measure the same underlying construct and evaluate the effects of both psychological distress in the context of other patient-related variables. Third, no other psychological profiles were analysed in this review apart from anxiety and depression. Consequently, this review is unable to comment on the influence of other important psychological factors such as emotions and control beliefs.

## Conclusion

In conclusion, pre-operative anxiety, depression, pain and poor function are significant predictors of a poor post-operative HRQoL. Given the substantial effect that pre-operative pain has on dissatisfaction, it is crucial for patients experiencing high levels of pain to be informed, of the chances of improvement by having a TKA. We recommend that patients are identified prior to surgery using a common psychological screening tool that separates depression and anxiety.

## Electronic supplementary material

Below is the link to the electronic supplementary material.

Supplementary material 1 (PDF 267 kb)

## Abbreviations

TKA: Total knee arthorplasty
QoL: Quality of life
HRQoL: Health-related quality of life
OA: Osteoarthritis
BMI: Body mass index
WOMAC: Western Ontario and McMaster University Osteoarthritis Index
HAD: Hospital Anxiety and Depression
KSS: Knee Society Score
VAS: Visual Analogue Score
OKS: Oxford Knee Score
KOOS: Knee Injury and Osteoarthritis Outcome Score
ROM: Range of motion
SF-36: Short Form-36
KSRS: Knee Society Rating System
STAI: State Trait Anxiety Inventory
DASS-A: Depression Anxiety Stress Scales Anxiety
BDI: Beck Depression Scale
SAI: State Anxiety Inventory
TAI: Trait Anxiety Inventory
BAI: Beck Anxiety Inventory
MCS: Mental Component Summary Scale
CASP: Critical Appraisal Skills Programme
