# Supplementary material for: Greater pre-operative anxiety, pain and poorer function predict a worse outcome of a total knee arthroplasty
Source: Knee Surg Sports Traumatol Arthrosc. 2016 Oct 12;25(11):3403–10. doi: 10.1007/s00167-016-4314-8 (PMC5644683; doi:10.1007/s00167-016-4314-8)
Supplement: Supplementary file 1 — Supplementary material 1 (PDF 267 kb) [file 167_2016_4314_MOESM1_ESM.pdf]

Supplementary Table 1

A list of search terms within each category used to identify all relevant papers

Category	Search terms used
1	Quality of Life; Outcome; QoL
2	Total Knee Replacement; Total Knee Arthroplasty; Total Knee Operation; TKA; TKR; Total Knee Surgery; Total Knee Prosthesis
3	Depression; Anxiety; Catastrophising

Supplementary Table 2- Summary of results from the Critical Appraisal Skills Programme Cohort Checklist

	CASP Checklist Question	**1**	**2**	**3**	**4**	**5**	**6**	**7**	**8**	**9**	**10**
Screening Questions	Did the study address a clearly focused issue?	√	√	√	√	√	√	√	√	√	√
	Was the cohort recruited in an acceptable way?	√	√	√	√	√	√	√	√	√	√
Detailed Questions	Was the outcome accurately measured to minimise bias?	√	√	√	√	√	√	√	√	√	√
	Have the authors identified all important confounding factors?	X	X	√	X	√	X	√	X	√	x
	Have they taken account of the confounding factors in the design and/or analysis?	X	X	√	√	X	√	X	X	√	X
	Was the follow up of subjects complete enough?	X	X	X	X	X	√	X	√	√	√
	Was the follow up of subjects long enough?	√	√	√	√	√	X	X	X	√	√
What are the results?	Are the results of the study clear?	√	X	√	X	X	√	X	X	√	X
	Are the results precise?	√	√	√	√	√	√	X	X	√	X
	Do you believe the results?	√	√	√	X	√	√	√	√	√	√
Will the results help locally?	Can the results be applied to the local population?	√	X	√	X	√	√	√	√	√	√
	Do the results of this study fit with other available evidence?	√	√	√	√	√	√	√	√	√	√

Supplementary Table 3- Summary of results that analysed depression as a predictive factor

Study	Depression	General outcome measures												
		Total WOMAC	WOMAC pain	WOMAC function	OKS	VAS pain	KOOS	KSS Joint	KSS function	KSS PAIN	KSRS	ROM	SF-36 PCS	SF-36 MCS
Blackburn et al.	HAD	*n/a*	*n/a*	*n/a*	√	*n/a*	*n/a*	*n/a*	*n/a*	*n/a*	*n/a*	*n/a*	*n/a*	*n/a*
Brander et al.	BDI	*n/a*	*n/a*	*n/a*	*n/a*	√	*n/a*	*n/a*	*n/a*	*n/a*	*n/a*	*n/a*	*n/a*	*n/a*
Duivenvoorden et al.	HAD	*n/a*	*n/a*	*n/a*	*n/a*	*n/a*	√	*n/a*	*n/a*	*n/a*	*n/a*	*n/a*	*n/a*	*n/a*
Hanusch et al.,	HAD	*n/a*	*n/a*	*n/a*	√	*n/a*	*n/a*	*n/a*	*n/a*	*n/a*	*n/a*	√	*n/a*	*n/a*
Hirschmann et al.	BDI	√	*n/a*	*n/a*	*n/a*	*n/a*	*n/a*	*n/a*	**X**	*n/a*	*n/a*	*n/a*	*n/a*	*n/a*
Lopez-Olivo et al.	DASS-D	√	*n/a*	*n/a*	*n/a*	*n/a*	*n/a*	*n/a*	*n/a*	*n/a*	√	*n/a*	*n/a*	*n/a*
Qi et al.	BDI	*n/a*	*n/a*	*n/a*	*n/a*	*n/a*	*n/a*	**X**	**X**	*n/a*	*n/a*	*n/a*	**X**	**X**
Noiseux et al.	GDS	*n/a*	*n/a*	*n/a*	*n/a*	*n/a*	*n/a*	*n/a*	*n/a*	*n/a*	*n/a*	**X**	*n/a*	*n/a*
Utrillas-Compaired et al.	HAD	√	*n/a*	*n/a*	*n/a*	**X**	*n/a*	*n/a*	√	**X**	*n/a*	*n/a*	*n/a*	*n/a*
Wylde et al.	HAD	*n/a*	**X**	**X**	*n/a*	*n/a*	*n/a*	*n/a*	*n/a*	*n/a*	*n/a*	*n/a*	*n/a*	*n/a*

Supplementary Table 4- Summary of results that analysed anxiety as a predictive factor

Study	Anxiety	General outcome measures												
		Total WOMAC	WOMAC Pain	WOMAC function	OKS	VAS pain	KOOS	KSS Joint	KSS FUNCTION	KSS PAIN	KSRS	ROM	SF-36 MCS	SF-36 PCS
Blackburn et al .	HAD	n/a	n/a	n/a	√	n/a	n/a	n/a	n/a	n/a	n/a	n/a	n/a	n/a
Brander et al.	STAI	n/a	n/a	n/a	n/a	√	n/a	n/a	n/a	n/a	n/a	n/a	n/a	n/a
Duivenvoorden et al.	HAD	n/a	n/a	n/a	n/a	n/a	**X**	n/a	n/a	n/a	n/a	n/a	n/a	n/a
Hanusch et al.,	HAD	n/a	n/a	n/a	√	n/a	n/a	n/a	n/a	n/a	n/a	**X**	n/a	n/a
Hirschmann et al.	STAI	√	n/a	n/a	n/a	n/a	n/a	n/a	√	n/a	n/a	n/a	n/a	n/a
Lopez-Olivo et al.	DASS-A	√	n/a	n/a	n/a	n/a	n/a	n/a	n/a	n/a	√	n/a	n/a	n/a
Qi et al.	BAI	n/a	n/a	n/a	n/a	n/a	n/a	**X**	**X**	n/a	n/a	n/a	√	**X**
	SAI	n/a	n/a	n/a	n/a	n/a	n/a	**X**	**X**	n/a	n/a	n/a	√	√
	TAI	n/a	n/a	n/a	n/a	n/a	n/a	**X**	√	n/a	n/a	n/a	√	**X**
Noiseux et al.	STAI	n/a	n/a	n/a	n/a	n/a	n/a	n/a	n/a	n/a	n/a	√	n/a	n/a
Utrillas-Compaired et al.	HAD	n/a	√	√	n/a	**X**	n/a	n/a	**X**	**X**	n/a	n/a	n/a	n/a
Wylde et al.	HAD	√	n/a	n/a	n/a	n/a	n/a	n/a	n/a	n/a	n/a	n/a	n/a	n/a

Supplementary Table 5- Summary of results that analysed 'age at surgery', 'gender', 'co-morbidities', 'BMI', 'level of education', 'pre-operative pain severity' and 'pre-operative knee function in the 10 included studies

Study	Predictive factors							
	Outcome measure	Age at surgery	Gender	Co-Morbidities	BMI	Level of education	Pre-operative pain severity	Pre-operative knee function
Blackburn et al .	OKS	*n/a*	*n/a*	*n/a*	*n/a*	*n/a*	*n/a*	*n/a*
Brander et al.	VAS Pain	*n/a*	*n/a*	*n/a*	*n/a*	*n/a*	*n/a*	*n/a*
Duivenvoorden et al.	KOOS	*n/a*	*n/a*	*n/a*	*n/a*	*n/a*	*n/a*	*n/a*
Hanusch et al.,	OKS	*n/a*	*n/a*	*n/a*	*n/a*	*n/a*	*n/a*	*n/a*
Hirschmann et al.	Total WOMAC	*n/a*	*n/a*	*n/a*	*n/a*	*n/a*	*n/a*	*n/a*
Lopez-Olivo et al.	WOMAC	**x**	√	√	√	√	√	√
	KSRS	**x**	√	√	√	√	√	√
Qi et al.	KSS	*n/a*	*n/a*	*n/a*	*n/a*	*n/a*	*n/a*	*n/a*
Noiseux et al.	ROM	**x**	**x**	*n/a*	**x**	**x**	√	*n/a*
Utrillas-Compaired et al.	KSS Function	√	*n/a*	√	√	*n/a*	*n/a*	√
	KSS pain	*n/a*	*n/a*	*n/a*	*n/a*	*n/a*	√	*n/a*
Wylde et al.	WOMAC pain	**x**	**x**	**x**	*n/a*	*n/a*	√	*n/a*
	WOMAC function	**x**	**x**	**x**	*n/a*	*n/a*	*n/a*	√
